# Cariprazine add-on in resistant bipolar depression. Long-term effectiveness and safety data from a multicentric real-world experience

**DOI:** 10.1192/j.eurpsy.2024.209

**Published:** 2024-08-27

**Authors:** V. Martiadis, E. Pessina, A. Martini, F. Raffone, A. Vignapiano, D. De Berardis

**Affiliations:** ^1^Department of Mental Health, Asl Napoli 1 Centro, Napoli; ^2^Department of Mental Health, Asl Cuneo 2, Bra; ^3^Department of Mental Health, First Episode Psychosis Center, Salerno; ^4^Department of Mental Health, Asl Teramo, Teramo, Italy

## Abstract

**Introduction:**

Persistent depressive episodes and subsyndromic depressive symptoms frequently characterize mood alterations in bipolar disorder (BD) and negatively influence quality of life and suicide risk. BD patients with predominant depressive episodes generally show significantly higher treatment resistance rates. Although not specifically approved in Italy for bipolar depression, recently published observational data suggest that the cariprazine add-on may be a potential effective short-term treatment for resistant bipolar depression. Nevertheless data on long-term cariprazine treatment are lacking.

**Objectives:**

This study evaluated the efficacy and safety of long-term cariprazine augmentation in patients suffering from treatment-resistant bipolar depression.

**Methods:**

30 resistant bipolar depressed patients, whose resistance was defined according to The CINP Guidelines on the Definition and Evidence-Based Interventions for Treatment-Resistant Bipolar Disorder, were treated with cariprazine 1,5 -3 mg flexible dose for 4 weeks, added to previous mood stabilizing and/or antidepressant treatment. Psychopathology at time 0 and at 4, 8, 12, 16, 20, 24 weeks of treatment was evaluated using the Hamilton Depression Rating Scale (HDRS), the Hamilton Anxiety Rating Scale (HARS), the Young Mania Rating Scale (YMRS) and the Bipolar Depression Rating Scale (BDRS); safety and tolerability was measured by the UKU Side Effect Rating Scale. The drop-out rate was assessed throughout the study duration.

**Results:**

Cariprazine add-on was effective in the study sample but only during the first 4 weeks of treatment. Improvement in depression scores started from the first week, reaching about 40% mean HDRS score reduction at T4; a moderate ulterior decrease (-15%) was reached at T24 but was accompanied by a significant drop-out rate; anxiety symptoms improved (mean HARS score reduction 37% at T4) mainly during the first 4 weeks. The treatment was generally well tolerated. From week 4 to 24 we observed a near 70% drop-out rate (18 total drop-outs) with maximum drop-outs between weeks 4-8 (n=7) and 18-24 (n=7). Discontinuation causes were inefficacy (5/18); clinical worsening (10/18); side effects (3/18); hypomanic shift (2/18).

**Conclusions:**

Despite the relatively small population examined and the observational design, our results suggest that cariprazine may represent an effective and safe short-term enhancement strategy in resistant bipolar depression. Long-term treatment, in this sample, did not lead to significant improvements and was burdened by a high drop-out rate, mainly due to inefficacy/clinical worsening. Further studies on larger samples are needed to confirm these preliminary findings, both in short-term and in longer observations.

**Disclosure of Interest:**

None Declared

